# Application of fuzzy multicriteria decision-making model in selecting pandemic hospital site

**DOI:** 10.1186/s43093-023-00185-5

**Published:** 2023-04-01

**Authors:** Alaa Alden Al Mohamed, Sobhi Al Mohamed, Moustafa Zino

**Affiliations:** 1 Department of Business Administration, Aleppo University, Aleppo, Syria; 2 Department of Business Administration, Ebla Private University, Aleppo, Syria

**Keywords:** TOPSIS, AHP, Fuzzy logic, Location, Uncertainty—multicriteria decision-making

## Abstract

One of the most important challenges for decision-makers and investors is location selection, which may be assessed using multicriteria decision-making (MCDM) methodologies. Problems with picking a location include deciding between alternative locations, analyzing alternatives, and identifying the best location for a hospital. Because they analyze options with multiple perspectives in terms of numerous competing criteria, MCDM approaches are useful instruments for solving decision-making challenges. The fuzzy set theory (FST), which represents uncertainty in human beliefs, may be effectively used with MCDM approaches to produce more sensitive, tangible, and accurate findings in this context. A hybrid fuzzy multi-criteria decision model (FMCDM) is proposed to find the optimal location based on a combination of factors. In the first stage, the Fuzzy Analytical Hierarchy Process (FAHP) is used to estimate the relative criteria classification through the evaluation process. In the second stage, the fuzzy technique of order preference using similarities to the perfect solution (FTOPSIS) is applied to rank the possible alternative sites. The findings from this study indicate that integrate FAHP and FTOPSIS is the most often used FMCDM approach in Aleppo for selecting the best location for a new hospital.

## Introduction

A decade after the beginning of the Syrian crisis in 2011, the country’s healthy sector is facing many challenges and obstacles where Many hospitals have been destroyed were forced to considerably reduce their operations as a result of the destruction of the infrastructure of most of the healthy sector and lack of resources [2]. Where the Syrian crisis has increased vulnerability and the prevalence of impairment, there are a tremendous number of war-born injuries that is considered as a problem in both sectors of health and socioeconomics. By the end of 2015, out of 113 assessed public hospitals under the Ministry of Health MoH and Ministry of High Education MoHE, 50% were reported fully functioning, 28% were reported partially functioning, while 28% were reported nonfunctioning as a result of its destruction; this shows the deterioration of the national health centers and the quality of services provided which increases the pace of the transition from injury to impairment and disability.

By the end of May 2018, the mentioned results of the situation of hospitals are almost the same [3]; this shows the deep damage left by the crisis. Access to health hospitals and the ability to provide required services who citizens face as a result of the crisis. Moreover, with the spread of the corona virus since its discovery near the end of 2019, in Wuhan, China, COVID-19 has expanded to several countries; COVID-19 has been declared an extraordinary health calamity and a worldwide epidemic by WHO. The COVID-19 epidemic has had a huge effect on people's lives, putting social life and public health in jeopardy and pushing economies into a tailspin [110].

All of that has created several issues for the health industry in general, and hospitals in particular, including a lack of supplies, equipment, human resources, and space, as well as placing strain on the whole healthcare system [62]. Each country has attempted various strategies to handle the challenges in its hospitals. During the epidemic, some countries, like as China and Turkey, erected emergency hospitals, while others, such as Spain, the USA, Brazil, and India, converted stadiums, dorms, and hostels into temporary pandemic hospitals, while in Syria, some hospitals have turned venues. With the deterioration of the Syrian situation and facing more challenges in general and in the health aspect in particular, the shortage of hospitals qualified to receive patients and with the high costs of treatment in the remaining private hospitals and the deterioration in the quality of health services provided, this is what prompted decision-makers in the health sector to contribute to the decision-making process, concerning identifying new sites for building new hospitals capable of solving even part of the problems faced by the health sector.

However, the decision-making process is a scientific way to assessing the possibilities for overcoming negative challenges that arise in the functioning of any institution and picking the best option among these options. When it comes to making decisions, the modern approach to operating a corporation necessitates the application of scientific methodologies that are upgraded and appropriate for today's situations. Making correct judgments necessitates a thorough understanding of the system in which issues occur, as well as the development of a trustworthy mathematical model (algorithm) that accurately depicts the problem or problems. The range of scientific methodologies utilized in decision-making processes is now seen to be the secret of business success. Furthermore, quick changes and competitive conditions make it necessary to work together to solve difficulties and to employ current technical procedures [37].

Site selection, which provides a systematic framework for establishing the norms and criteria for the geographical placement of production, commerce, and service activities, is relevant in this context and has been identified as a key research theme, attracting a growing number of researchers and decision-maker from numerous scientific disciplines. Furthermore, because of its considerable and long-term implications on commercial risks, expenses, and revenues, site selection is one of the most essential strategic business choices [58]. Selecting a particular hospital location requires considering many potential criteria, such as investment cost, human resources, availability of acquirement material, and market condition [104].

Multiobjective decision-making and multiattribute decision-making (MADM) are two of the model's issues [49]. Because it involves concerns from several domains and there are multiple, often opposing stakeholders to consider, hospital site selection can be regarded as a multi-criteria decision-making (MCDM) problem [27]. This paper introduces a multi-criteria decision-making (MCDM) method for selecting the optimum option from a set of options by weighing many criteria [56].

The multi-criteria decision-making (MCDM) approach, which encompasses several approaches, has been used to examine the bulk of site selection difficulties like analytical hierarchical process (AHP), Analytical Network Process (ANP), Decision Making Trial and Evaluation Laboratory (DEMATEL), Technique for Order Preference by Similarity to an Ideal Solution (TOPSIS), VIsekriterijumska optimisacija I KOmpromisno Resenje (VIKOR), and others. Soft computing techniques including Multi-Objective-Decision-Making (MODM), fuzzy sets, the analytic method, mixed integer/linear integer/goal programming, cluster analysis, and others were also applied [60].

Although there are many evaluation techniques, the MCDM model is a well-liked strategy that is successfully applied in many sectors for optimal site decision-making under several competing criteria. This approach is crucial in management science, which can improve service quality and aid in hospital ranking [105]. Due to their ability to resolve complicated problems that concurrently signify qualitative and quantitative evaluations of the criteria, two strategies are typically utilized in MCDM-based methods to construct a more accurate decision-making structure to evaluate and choose the ideal site [18, 101]. The analytical hierarchy process (AHP) and the TOPSIS technique for ranking preferences according to how closely they resemble the ideal answer are two among them [67]. The following list outlines the overall structure of studies using the MCDM approach. First, the alternative possibilities and the standards for judging them are put forward. Then, using the AHP technique, the criteria weights against choices are determined based on the influent coefficients of each criterion in contrast to others. Finally, these criteria weights can be used using TOPSIS [94] to rank alternative solutions. Additionally, the combination of fuzzy AHP and fuzzy TOPSIS has developed into a powerful tool for combining several options into the choice that meets the requirements the best [81]. The fuzzy approach makes it easier and more accurate to calculate the score of the criteria weights than previous methods do. Only experts or managers need to perform qualitative evaluations. Fuzzy sets are used to digitize the relative influence of one criterion on another, while the criteria are compared in pairwise comparisons. As a result, this approach can aid experts and investors in avoiding ambiguity and confusion during decision-making [75]. Numerous studies based on the merging of two AHP and TOPSIS methodologies in the fuzzy environment have been carried out to address difficult issues.

In fuzzy MCDM methods, the main reason of using FTOPSIS as basic technique is that fuzzy TOPSIS method has been used to address a variety of matters, including facility selection [21], choosing between computer-assisted manufacturing systems [9], plant location selection [104] measuring quality of service in the hotel industry for selecting priority services [8], evaluating initial training airplanes under a fuzzy environment [95], Weapon selection [24], suppliers selection [61, 73], Location Selection of Shopping Malls [37], in rising economies, choosing a bearable site for a waste electrical and electronic equipment recycling facility [60] as shown in Table 1.

**Table 1:** Applications of fuzzy TOPSIS

Application	Contributor(s)
Evaluation of airline performance	Wang [96]
Bridge risk assessment	Wang and Elhag [97]
Evaluating environmental performance of suppliers	Lin and Chang [65]
Considering the advantages of shopping websites in terms of competition	Sun and Lin [91]
Evaluating performance of traffic police centers	Sadi-Nezhad and Khalili Damghani [84]
Evaluating software development projects	Büyüközkan and Ruan [10]
Initial training aircraft	Wang and Chang [95]
Inter-company comparison	Deng et al. [28]
Marketing examination and selection of middlemen	Dikmen and Say [30]
Measuring flexibility of computer integrated manufacturing systems	Kahraman et al. [48]
New product introduction	Liao et al. [64]
Personnel selection	Kelemenis and Askounis [55]
Selection of planning and design tenders in public office buildings	Hsieh et al. [44]
Plant layout design problem	Yang and Hung [101]
Plant location selection	Yong [104]
Project risk assessment	Ebrahimnejad et al. [34]
Oil-field development project selection	Amiri [5]
Deployment of quality functions	Kavosi and Mavi [53]
Rapid prototyping process selection	Byun and Lee [11]
Robot selection	Dutta and Borah [33]
Strategic management decisions	Emami et al. [36]
Green Supplier Selection	Doğan et al. [31]
Trans-shipment site selection	Önüt and Soner [72]
Vessel selection problem	Yang et al. [102]
Warehouse location selection	Ashrafzadeh et al. [6]

In the tourist sector, Baki et al. recommended a fuzzy AHP-TOPSIS model to increase hotel website operating efficiency by boosting competition, brand value, and consumer volume [75]. Given the priority solutions for reverse logistics barriers that were also implemented by the same tools, this can assist in identifying the best third-party logistics (3PL) for cold chain management [13, 86]. The fuzzy AHP-TOPSIS model also looked at the other issues, such evaluating sustainable urban development in an emerging economy. Additionally, the other aspects were investigated by this fuzzy AHP-TOPSIS model, such as assessing sustainable urban development in an emerging economy [25], evaluating the maintenance factors that affected sustainable manufacturing [46]. According to a review of the above studies, it can be seen that the hybrid MCDM method was effectively applied in decision-making regarding the optimum site selection in varied sectors.

However, the related literature on choosing the most potential site of hospital in Aleppo is unavailable, and this has not been fully researched and exploited to date. For this reason, the Fuzzy AHP and Fuzzy TOPSIS method are integrated to address our concerns regarding this problem. The study aims to efficiently assist investors or decision-makers in the evaluation and selection of suitable hospital locations in Aleppo. The literature procedure is organized as follows. Firstly, an evaluation of the criteria weightings is conducted using the fuzzy AHP method based on experts’ opinions combined with a literature review. Next, the alternatives to the healthy site are ranked by applying fuzzy TOPSIS. The model’s result assists investors in further development, based on the priority ranking of the hospital sites.

Moreover, strengthening these results using sensitivity analysis and review 12 cases in which the weights (input data) were changed, the results were drawn, and the main results extracted from the use of the fuzzy TOPSIS technique were strengthened and contribute to providing a framework for decision support to address the problem of choosing the location of the temporary hospital in a cloudy environment of uncertainty. We presented a generalization of TOPSIS in an ambiguous environment and the use of the proposed technique in selecting the optimal site for hospital construction in Aleppo, a Syrian governorate according to a set of criteria.

The remaining portions of the paper are divided into five categories. The literature review examines in the next section. In section "Methodology" describes the methodology, and the results and discussion section describes in the section "Results and discussion." The paper comes to a close with the conclusion.

## Literature review

This section reviews prior research on selecting optimal location for building hospitals in Aleppo built on a set of criteria. The primary objective of this research is to develop a mathematical model capable of dealing with incomplete or inaccurate data and arriving at suggested solutions in which the decision-maker is an essential part of the decision-making process. There are three sections to this literature review. The first portion goes through the fundamentals of decision making and basic requirements for decision making. The second section discusses the process of location selection. Finally, the importance of multi-criteria decision-making (MCDM) is discussed in the last one.

### MCDM literature

An overview of the main characteristics of MCDM methods and challenges is given in this subsection.

A typical MCDM challenge entails selecting the best option from a set of workable options that have been assessed using a variety of competing quantitative and qualitative criteria. Assume a decision-maker (DM) is asked to rank n alternatives (also known as options or choices), $i.e.,A1,A2,A3,...,An$, according to m criteria, $i.e., C1,C2,C3,...,Cm$. Assuming that j is the relative weight of *Cj*, with $j>0$ and $mj=1j=1$, let $xij$ be the score of $Ai(i={1,2},...,n)$ on the criteria $Cj(j={1,2},...,m)$. Now, the decision matrix below can be used to represent this MCDM problem.

The problem definition, alternative selection, criterion selection, decision matrix creation, weight elicitation (determining the weight of the criteria), and alternative ranking are generally the primary steps of MCDM techniques. The decision criteria are frequently divided into two categories: benefit criteria, where the performance improves with increasing alternative score (for example, profit), and cost criteria, where performance improves with decreasing alternative score (e.g., price) [22, 71, 79, 88, 103, 107].

Well-known and traditional MCDM techniques including WSM, AHP, ANP, WPM, TOPSIS, VIKOR, ELECTRE, PROMETHEE, GRA, and DEMATEL can be used to solve MCDM problem. Additionally, a lot of academics have tried to create new MCDM techniques to address problems in the real world with various features, such as COPRAS, WASPAS, BWM, SWARA, MULTIMOORA, SODOSM, ARAS, OPA, MARCOS, and GLDS.

Around 100 MCDM approaches (with variations) have been documented in the literature, and Greco et al. (Greco et al. 2016) noted that DMs can be unsure of which one to use. The kinds of issues that MCDM approaches seek to solve, the theoretical foundations they are based on, and the kinds of conclusions they draw are all different [74, 87]. As a result, they were developed to deal with issues of various types and levels of complexity [4]. For instance, PROMETHEE has assumed a significant position among the MCDM techniques [26]. revealed that this strategy can only be employed provided several crucial considerations are made. Dožić noted that the appropriate application of each MCDM technique depends on the problem at hand and the availability of necessary data [32]. As a result, no single MCDM approach can be employed to solve all kinds of decision issues [43, 57, 88, 103, 106].

On the other hand, Gershon and Duckstein highlighted the main criticism of MCDM methods, pointing out that it is generally difficult to determine which MCDM method is more reliable for a given problem and that different methods may produce different results when applied to a specific decision problem [40]. More importantly, there is still debate over the accuracy of the ranking results [103]. Of course, in some circumstances, making the wrong choice could result in significant or irreversible losses. In fact, the abundance of accessible approaches might be confusing for DMs, who then have the difficult problem of deciding which MCDM method is best [106]. Subsequently, many academics have offered recommendations for choosing an acceptable MCDM approach (e.g., (Coello and Jin, n.d., [70, 74, 79, 80], while others have applied multiple MCDM approaches to a specific issue and contrasted the outcomes [70, 88]. For instance, Chen and Pan [35] presented a two-step strategy to pick appropriate fuzzy MCDM methods for tackling construction management problems to overcome this issue. To choose a good MCDM approach for a particular decision problem, however, is a substantial research challenge that has not yet been solved, according to the literature [98].

The strengths and weaknesses of MCDM approaches for certain applications are well-documented in the literature. [88], for instance, evaluated the advantages and disadvantages of various MCDM techniques for the optimal search issue. The benefits and drawbacks of fuzzy MCDM techniques in construction management were described by Chen and Pan [17]. In order to solve the problem of material selection, Mousavi-Nasab and Sotoudeh-Anvari [70] investigated the characteristics of MCDM approaches. The choice of a particular MCDM approach appears to be motivated by familiarity and/or affinity, according to Cinelli et al. [22], who noted that many writers do not adequately explain the justifications for selecting one MCDM method over another. Simply put, MCDM techniques are widely used today due to the availability of numerous software packages. However, according to Hobbs and Horn [43], if general DMs do not understand how an MCDM method works, they may not trust the obtained results.

We are aware that there are various approaches to classify MCDM techniques. Multi-attribute utility theory (MAUT) techniques like TOPSIS and VIKOR and outranking techniques like ELECTRE and PROMETHEE can be used to categorize MCDM techniques as a whole [108]. A pairwise comparison between alternatives for each criterion forms the basis of an outranking approach, and the outranking relations are formed by aggregating the pairwise comparisons [108]. The following phases are frequently used in MAUT methods: creating a decision matrix, normalizing it, adding up each option's performance value in relation to each choice criterion, and rating the options [79, 99, 108] are the first three steps. Consequently, the key reason why various MCDM methods may generate different outcomes lies in the diversity of normalization methods and aggregation functions employed [43, 57, 88, 103, 106].

Everyone is aware that several units analyze how alternatives perform in relation to the choice criteria. To make several scales of measurement comparable, normalization procedures are used to the components of the provided decision matrix. There are various normalization approaches, and each one has its own advantages, disadvantages, and focal points [69]. Additionally, various normalization methods could produce various results [99]. According to Milani et al. [69], who examined the effects of various normalization procedures in MCDM methods, normalization inside an MCDM method may not result in a choice that is reliable. However, choosing a good normalizing strategy for an MCDM method has received little attention [50].

Regarding identifying places and locations, the TOPSIS mysterious method is considered one of the most prominent methods that have been used in this field. There are many previous studies, including [37, 38], Kasim, n.d.; [85, 92] all of which applied this method in determining the most appropriate location among the existing alternatives.

The previously mentioned multi-criteria decision-making (MCDM) methods are some of the frequently used decision-making tools that are well designed for complex problems involving multiple criteria with the need to prioritize alternatives. MCDM methods enable the decision-makers to evaluate the number of alternatives with the different data available such as crisp, fuzzy, interval, rough, etc. The criteria importance through inter-criteria correlation (CRITIC) approach is a well-known MCDM method, which is designed to determine the criteria's importance to an MCDM problem. Unlike other MCDM methods that focus on the weighing process, the CRITIC method does not require separate pairwise comparisons of criteria, because it uses an initial decision matrix that is generated to compare the alternatives, as one of the powerful MCDM methods presented recently.

### Decision making (DM) and its requirements

Decision making is a scientific way to assessing the possibilities for overcoming negative challenges that arise in the administration of any organization and choosing the greatest alternative among these possibilities.

MCDM is a methodological frame that tries to suggest decision-makers with informed recommendations from a finite number of options (also known as actions, solutions, or candidates) that have been examined from numerous perspectives, or criteria (sometimes known as characteristics, qualities, or goals). The problem of selecting location is commonly referred to MCDM in the literature. As a result, traditional MCDM techniques have been used in problem-solving processes. MCDM techniques can be divided into four groups based on the principles that underpin them:(1) "multiattribute utility methods like AHP and ANP, (2) outranking methods like Elimination and Choice Expressing Reality (ELECTRE) and Preference Ranking Organization Method for Enrichment Evaluation (PROMETHEE); (3) compromise methods like Technique for Order Performance by Similarity to Ideal Solution (TOPSIS) and Multicriteria Optimization and Compromise Solution (VIKOR); and (4) other MCDM techniques like Simple Multiantibiotic Rating Technique (SMART) (DEMATEL)” [14], but in other hand, to overcome ambiguity in decision-making difficulties, MCDM approaches can be combined with fuzzy logic. In the literature, there are several kinds of fuzzy MCDM methods and their applications. Fuzzy MCDM approaches are used to handle energy policy and decision-making challenges [54].

### Construction of hospitals and its importance in facing corona

Hospitals all throughout the world are critical in the fight against the COVID-19 epidemic. During an epidemic, hospital executives are usually confronted with unexpected problems that need them to undertake jobs they are not used to [1].

As the current coronavirus outbreak has demonstrated, many contemporary hospitals and healthcare systems lack the flexibility to handle unanticipated increases in patient numbers. Hospitals have reached capacity to treat COVID-19 patients suffering from extreme symptoms while also caring for individuals with mild symptoms or no symptoms, who pose an infectious danger to healthcare personnel and other patients.

To address this issue, hospital architects have discovered new design options for healthcare systems, which have traditionally been built to support lean and efficient care operations, with an emphasis on delivering health services closer to patients' homes, inside community settings. In regard to their local surroundings, healthcare organizations and hospitals have three main options for transforming their environments in response to COVID-19.

Spatial and organizational improvements have been implemented to better accommodate COVID-19 patients within a hospital, including the restructuring of internal 'foot traffic' routes and flows to separate persons infected with coronavirus from those who are not.

For example, in Syria, the government team held a meeting in which it evaluated the decisions taken to strengthen the health work system in the country and to prepare hospitals to receive cases infected with the coronavirus, where the necessary measures were taken to ensure the increase of artificial respirators, intensive care beds, ambulances and medical equipment, and to secure their supplies of fuel and maintenance. In addition, December 16 in Damascus, Syrian health officials have activated an emergency hospital that was recently created to deal with an influx of patients that was established at a tennis court inside the Faiha sports stadium north of Syrian capital Damascus, the emergency hospital is equipped with 120 beds, 300 oxygen cylinders as well as 50 Continuous Positive Airway Pressure (CPAP) machines, in addition to a qualified and trained medical team to receive the COVID-19 cases.

## Methodology

### Aggregate the weight of criteria

Criteria weights are important in multi-criteria decision-making methods. Although these weights carry many different meanings [19], these weights can influence the final decision especially in evaluation or selection problems. There are many methods available for determining weights, but the methods are mostly categorized into two main approaches: subjective and objective methods. Some researchers [66, 68] have used aggregated weights that combine subjective and objective weights to balance the evaluator(s) and data-driven impact. Subjective methods involve the rater(s) of different backgrounds and experiences, and the analytical hierarchy process (AHP) is one of the most popular Subjective methods developed by [82], and Full Consistency Method (FUCOM), the Best Worst Method (BWM), along with issues of inconsistency and the problem of rank inversion. The most classic methods are the classification method and the direct assignment of points [78], the ratio method [35] and the swing method. Among the rank-based methods [7] are rank-sum (RS), rank reciprocal (RR) and rank-centroid (RC) methods, while objective weights are found by processing intrinsic information in the criteria, to avoid human autonomy, researchers can choose methods for weighting objective criteria where these methods are of the data-driven type. Among the methods are entropy [109], the importance of criteria through common criteria (CRITIC) by [29] standard deviation and coefficient of variation [51], there are fuzzy measures where subjective and objective methods as discussed in the previous two sections focus on the individual weights of the criteria. However, the concept of fuzzy measures is associated with compound weights. Besides considering the weights of individual criteria, these fuzzy scales represent the measures of interaction between criteria or between criteria introduced by [89], one type of fuzzy measure is called as λ-fuzzy measure [51], and another type is called as k0-measure [59].

The MCDM technique, which is suggested to handle complex decision issues, includes the analytical hierarchy process (AHP) as a useful tool [82]. With this approach, the structure is described at various element levels, including criteria, subcriteria, and alternatives, and a comparison is made between them [83]. The outcomes are influenced by the decision-makers' perspective on the numbers in real-world scenarios, and the AHP approach is unable to solve a severely unbalanced scale of judgment with certainty [90]. This method is further enhanced by the fuzzy AHP, which extends the AHP [93]. Due to the simplicity with which thoughts can be expressed, this evaluation is more practical and relevant than conventional methods [20]. The fuzzy AHP method is therefore commonly applied in reality. The most appropriate Supplier is picked to meet the unique requirements of the airline retailing business, which is a very complicated notion. Using the fuzzy AHP technique, an effective system in terms of both quantitative and qualitative decision variables is constructed to select the worldwide vendor [77]. In a similar vein, this approach is used to determine where a warehouse should be located in order to maximize supply chain efficiency. Additionally, the fuzzy AHP tool resolves sustainability challenges while making judgments about strategic planning and management in both industry and society [12]. Specifically, it was shown that the proposed model for fuzzy AHP technique measures the weights of the criteria within the seventh division.

### Fuzzy set

The complication of the system on which judgments will be made can sometimes rise, the significance of the ideas used to characterize that system can sometimes diminish, and there is a trend toward uncertainty. "Fuzziness" refers to the uncertainty of the terms used to describe an aim and a system. People's differing mental systems and perceptions might be seen as a cause of "fuzziness." There may be ambiguities due to under-maturation of human thinking, uncertainty, or "fuzziness" in some circumstances. In certain cases, numerical values cannot be utilized as decision-making input; instead, linguistic variables are employed, and fuzzy logic conditions are incorporated in the process.

### Fuzzy logic

The characteristic function is generalized by fuzzy sets, which accept all values between zero and one. membership function (a generalization of the characteristic function) defines a fuzzy subset $F$ of $X$, also written $F(x)$, whose values can be any number between 0 and 1. The value of F(x) is called the grade of membership of x in fuzzy set $F$, and it is frequently expressed by $\mu (x)$. If $\mu (x)$ is only zero or one, then we get the characteristic function of a crisp, nonfuzzy, set $F$. Now suppose we have $\mu (x)$ taking on values in [0,1] besides just zero and one. We say $x$ belongs to F if ${\mu }_{F} (x) = 1$, x does not belong to $F$ when ${\mu }_{F} (x)= 0$, and $x$ is in F with membership ${\mu }_{F} (x)$ if $0 <{\mu }_{F} (x) < 1$. The universal set always has ${\mu }_{X} (x) = 1$ for all x in X, and the empty set is described by its membership function always zero $[ {\mu }_{O} (x) = 0 for all x in X$]. Crisp sets are considered special cases of fuzzy sets when membership values are always 0 or 1 [55].

### Making uncertain decisions

The multi-criteria decision-making (MCDM) process benefited greatly from the fuzzy sets theory, and decision analysis has emerged as one of the most appropriate applications for this theory. Fuzzy MCDM was created, which marked a significant improvement in the field of MCDM by incorporating fuzzy sets into the MCDM procedure. Although the weights and priorities of the criteria are assumed to be precisely understood by the classical MCDM approaches, they fall short in modeling situations that are experienced in reality. In addition to permitting the use of linguistic factors in evaluating criteria and alternatives, fuzzy MCDM approaches also produce successful outcomes by quantifying unclear data.

Many times, the data used in the decision-making process are incomplete or unreliable. When decision-makers must make a variety of decisions based on uncertain and ambiguous facts, fuzzy sets theory should be used. Furthermore, when solving real-world decision-making issues with fuzzy sets, the outcomes are more realistic. The main benefit of fuzzy decision making is that it provides a structure with more flexibility for coping with issues resulting from a lack of information [42]. Similarly, utilizing a fuzzy approach to decision-making has the advantage of representing relative characteristic prioritizing with fuzzy numbers rather than precise values.

### Fuzzy numbers

#### Definition 2.1

("*Fuzzy set*") Assume $X$ is a discourse universe, where $\widetilde{A}$ is a fuzzy subset of$X$; and for all $x\in X$, there is a number${\mu }_{\widetilde{A}}\in \left[{0,1}\right]$, The membership of $\widetilde{A}$ is the term used to describe the membership of x in$\widetilde{A}(\mathrm{Yong}, 2006)$.

#### Definition 2.2

("*Fuzzy number*") A fuzzy number $\widetilde{A}$ is a fuzzy subset of $X$ that is both normal and convex. "Normality" indicates in this case that:

$$\exists x\in {\mathbb{R}},\bigvee {\mu }_{\widetilde{\mathrm{A}}}\left(\mathrm{x}\right)=\mathrm{i}1$$

And “convex: means that:

$$\forall {\mathrm{x}}_{1}\in \mathrm{X}, {\mathrm{X}}_{2}\in \mathrm{X}, {\forall }_{\alpha } \in \left[\mathrm{0,1}\right]$$

$${\mu }_{\widetilde{A}}(\left(a{x}_{1}+\left(1-\alpha \right){x}_{2}\right)\ge \mathrm{min}({\mu }_{\widetilde{A}}\left({x}_{1}\right), {\mu }_{\widetilde{A}}\left({x}_{2}\right))$$

#### Definition 2.3

**Fig. 1:**
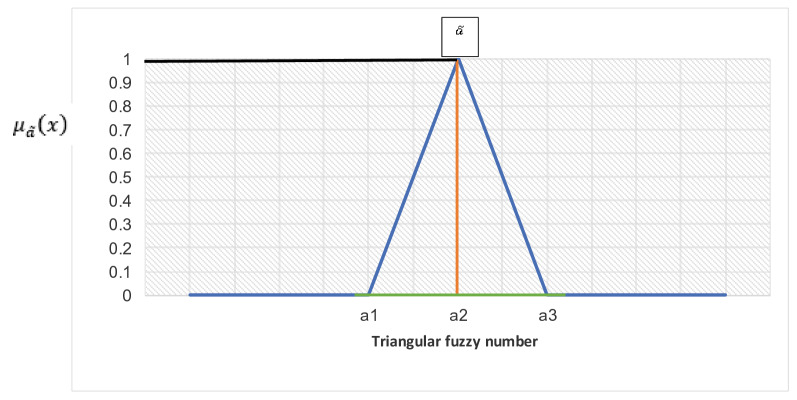
The membership functions

A triplet $({a}_{1}, {a}_{2}, {a}_{3})$ can define a triangular fuzzy number, as shown in Fig. 1.

The membership function ${\mu }_{a}$(x) of triangular fuzzy number $\widetilde{a}$ is provided by:

1 $${\mu }_{a}\left(x\right)= \left\{\begin{array}{c}0 : x<{a}_{1}\\ \frac{x-{a}_{1}}{{a}_{2}-{a}_{1}}: {a}_{1}\le x\le {a}_{2}\\ \frac{{a}_{3}-x}{{a}_{3}-{a}_{2}}: {a}_{2}\le x\le {a}_{3}\\ 0 : x>{a}_{3}\end{array}\right.$$

where ${a}_{1}, {a}_{2} ,{a}_{3}$ are real numbers, and${a}_{1} < {a}_{2} < {a}_{3}$. The highest grade of ${\mu }_{\widetilde{a}}(x)$ is given by the value of x at${a}_{2}$, i.e.,${\mu }_{\widetilde{a}}(x)=1$; it is the most likely value of the assessment data. The lowest grade of${\mu }_{\widetilde{a}}(x), i.e., {\mu }_{\widetilde{a}}(x) = 0$, corresponds to the worth of x at${a}_{1}$; it is the least likely value of the assessment data. Constants and ${a}_{1}, {a}_{2},{ a}_{3}$ are the lowest and upper boundaries of the accessible area for the evaluation data [63]. The fuzziness of the evaluation data is reflected in these constants (Fig. 1).

#### Property 1

Given two fuzzy triangular numbers $\widetilde{a}=\left({a}_{1},{a}_{2},{a}_{3}\right)$ and $\widetilde{b}=\left({b}_{1},{b}_{2},{b}_{3}\right)$, the main operations are expressed as follows:

1. Adding two triangular fuzzy numbers:
  2 $$\widetilde{a}+\widetilde{b}=\left({a}_{1}+ {b}_{1} ,{a}_{2}+ {b}_{2} ,{a}_{3}+ {b}_{3}\right), {a}_{1}\ge 0, {b}_{1}\ge 0$$
2. Multiplication of two triangular fuzzy numbers:
3. Subtraction of two triangular fuzzy numbers:
  3 $$\widetilde{a}*\widetilde{b}=\left({a}_{1}* {b}_{1} ,{a}_{2}* {b}_{2} ,{a}_{3}* {b}_{3}\right), {a}_{1}\ge 0, {b}_{1}\ge 0$$
  4 $$\widetilde{a}-\widetilde{b}=\left({a}_{1}- {b}_{1} ,{a}_{2}- {b}_{2} ,{a}_{3}- {b}_{3}\right), {a}_{1}\ge 0, {b}_{1}\ge 0$$
4. Division of two triangular fuzzy numbers:
  5 $$\widetilde{a}(/)\widetilde{b}=\left({a}_{1}/ {b}_{1} ,{a}_{2}/ {b}_{2} ,{a}_{3}/ {b}_{3}\right), {a}_{1}\ge 0, {b}_{1}\ge 0$$
5. Inverse of a triangular fuzzy number:
  6 $${\widetilde{a}}^{-1}= \left({1/a}_{1},1/{a}_{2},1/{a}_{3}\right)$$
6. Symmetric image:
  7 $$\widetilde{a}=\left(-{a}_{1},-{a}_{2},-{a}_{3}\right)$$

#### Property 2

**Fig. 2:**
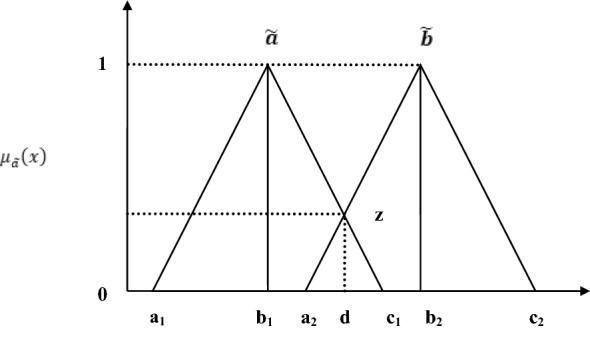
The intersection between $\widetilde{{\varvec{a}}}$ and $\widetilde{{\varvec{b}}}$

Given two triangular fuzzy numbers $(\widetilde{a}; \widetilde{b})$ and any real number $k$, the commutative operations of these two numbers are expressed as follows (Fig. 2):

Tables 2 and 3, which define a concept of fuzziness degree and formulate two criteria for the linguistic scale, are where the main contribution to the problem of evaluating the linguistic scale quality was produced.

**Table 2:** Linguistic scales of criteria ratings for FAHP

Linguistic term	Fuzzy number	Triangular fuzzy scale			Reverse triangular Fuzzy number		
Equal	$\widetilde{1}$	$1$	$1$	$1$	$1$	$1$	$1$
Weak advantage	$\widetilde{2}$	$1$	$2$	$3$	$1/3$	$1/2$	$1$
Not bad	$\widetilde{3}$	$2$	$3$	$4$	$1/4$	$1/3$	$1/2$
Preferable	$\widetilde{4}$	$3$	$4$	$5$	$1/5$	$1/4$	$1/3$
Good	$\widetilde{5}$	$4$	$5$	$6$	$1/6$	$1/5$	$1/4$
Fairly good	$\widetilde{6}$	$5$	$6$	$7$	$1/7$	$1/6$	$1/5$
Very good	$\widetilde{7}$	$6$	$7$	$8$	$1/8$	$1/7$	$1/6$
Absolute	$\widetilde{8}$	$7$	$8$	$9$	$1/9$	$1/8$	$1/7$
Perfect	$\widetilde{9}$	$8$	$9$	$10$	$0$	$1/9$	$1/8$

**Table 3:** Linguistic terms for alternative ratings

Linguistic term	Membership function
Very low (VL)	**(0,0,0.1)**
Low (L)	**(0,0.1,0.3)**
Mid Low (ML)	**(0.1,0.3,0.5)**
Medium (M)	**(0.3,0.5,0.7)**
Mid High (MH)	**(0.5,0.7,0.9)**
High (H)	**(0.7,0.9,1.0)**
Very High (VH)	**(0.9,1.0,1.0)**

Which The linguistic scale should introduce the least amount of fuzziness possible throughout the process of professional parameter evaluation. In addition, the linguistic scale should ensure that the data gathered from many specialists are as consistent as possible.

In Table 2, the linguistic variables that were used to evaluate the criteria for FAHP are shown, along with the triangular fuzzy numbers that correspond to them [76, 100], while Table 3 reports the linguistic variables that were used to evaluate the alternatives, along with the triangular fuzzy numbers that correspond to them [15]; it could be expressed in Table 2, 3:

#### Property 3

Let $\widetilde{a}=\left({a}_{1},{a}_{2},{a}_{3}\right)$ and $\widetilde{b}=\left({b}_{1},{b}_{2},{b}_{3}\right)$ be two fuzzy triangular numbers.

The distance between them using the vertex method is given by:

8 $$d\left(\widetilde{a}; \widetilde{b}\right)= \sqrt{\frac{1}{3}\left[{\left({a}_{1}- {b}_{1}\right)}^{2}+{\left({a}_{2}- {b}_{2}\right)}^{2}+ {\left({a}_{3}- {b}_{3}\right)}^{2}\right]}$$

### Linguistic variable

When dealing with situations that are too complicated or poorly defined to be fully represented using standard quantitative words, the concept of a linguistic variable comes in aid. Each linguistic value is represented by a fuzzy set of values, which are expressed as words, phrases, or artificial languages [27]. The importance weights of numerous criteria, as well as the evaluations of qualitative criteria, are used as linguistic variables in this paper. Positive triangular fuzzy numbers can be used to express these linguistic variables, as shown in Tables 2 and 3.

### Fuzzy analytical hierarchy process (FAHP)

**Step 1:** Pairwise comparison matrices of criteria.

Assume that a decision group will invest in this project with the help of K experts who will make the decision. Pair-wise comparison matrices are created among all the requirements of the hierarchical structure by determining which of the two dimensions is more crucial, according to the following formula matrix (${\widetilde{u}}^{k})$:

9 $${D}_{1}=\left[\begin{array}{ccc}1& {\widetilde{u}}_{12}^{k}& \begin{array}{ccc}{\widetilde{u}}_{13}^{k}& {\widetilde{u}}_{14}^{k}& \dots {\widetilde{u}}_{1n}^{k}\end{array}\\ {\widetilde{u}}_{21}^{k}& 1& \begin{array}{ccc}\cdots & \cdots & \cdots {\widetilde{u}}_{2n}^{k}\end{array}\\ \begin{array}{c}\vdots \\ \vdots \\ \dots {\widetilde{u}}_{n1}^{k}\end{array}& \ddots & \begin{array}{c}\begin{array}{ccc}1& \cdots & \cdots \end{array}\\ \begin{array}{ccc}..& 1& ..\end{array}\\ \begin{array}{ccc}\dots & \dots & 1\end{array}\end{array}\end{array}\right]$$

where ${\widetilde{u}}_{ij}^{k}$ is the fuzzy comparison value by kth decision-makers from the $ith$ to $jth$ criterion.

**Step 2:** Fuzzy geometric mean and fuzzy criteria weightage.

Determine the fuzzy geometric mean and fuzzy weights of each criterion based on the geometric technique introduced by [44], using following formulas, respectively.

10 $${\widetilde{r}}_{i}={\left({\widetilde{u}}_{i1}\otimes \dots \otimes {\widetilde{u}}_{ij}\dots \otimes{\widetilde{u}}_{in}\right)}^{1/n}$$

11 $${\widetilde{w}}_{i}={{\widetilde{r}}_{i}\otimes \left({\widetilde{r}}_{1}\oplus \dots \oplus {\widetilde{r}}_{i}\dots \oplus {\widetilde{r}}_{n}\right)}^{-1}$$

where ${\widetilde{u}}_{ij}=\frac{{\sum }_{k=1}^{k}{\widetilde{u}}_{ij}^{k}}{k}$ is the integrated fuzzy comparison value by the kth decision-maker from the $ith$ to $jth$ criterion, ${\widetilde{r}}_{i}$ is fuzzy geometric mean of the $ith$ criterion, ${\widetilde{w}}_{i}$ is fuzzy weight of the $ith$ criterion.

**Step 3:** BNP value for rating weight.

To assess the rating weights of criterion, compute the best nonfuzzy performance (BNP) value using following Formula:

12 $${BNP}_{wi}=\frac{\left[\left({u}_{wi}-{L}_{wi}\right)+\left({w}_{wi}-{L}_{wi}\right)\right]}{3}+{L}_{wi}$$

where ${BNP}_{wi}$ is the best nonfuzzy performance value, and $({U}_{wi}, {W}_{wi},{ L}_{wi})$ are the upper, middle and lower values of criteria weight.

### Fuzzy TOPSIS (FTOPSIS)

Hwang proposed Technique for Order Preference by Similarity to Ideal Solution (TOPSIS) approach is one of the most well-known multiple criterion decision-making (MCDM) methods. To identify the optimal option, the TOPSIS technique uses the shortest distance from the positive ideal solution (PIS) and the farthest distance from the negative ideal solution (NIS). While the PIS aims to maximize benefits while minimizing costs, the NIS aims to maximize costs while minimizing benefits.

To use the TOPSIS approach, there must be at least two possibilities, which necessitates first identifying the criteria. TOPSIS uses two sorts of criteria in general: “benefit criteria” and “cost criteria”[45]. In the cost criteria, the best value is the least (lowest cost), whereas in the benefit criteria, it is the greatest value (the most beneficial). To put it another way, the worst value in the cost criteria is the highest (the greatest cost), while the smallest value in the benefit criteria is the lowest (the least beneficial) [39]. The procedure of the fuzzy TOPSIS method is stated as follows:

**Step 1:** Assignment of ratings to the criteria and the alternatives.

Let us assume there are $J$ possible candidates called $A =\{A1, A2, . . . , Aj\}$ which are to evaluated against $n$ criteria,$C = \{C1, C2, . . . , Ci\}$. The criteria weights are denoted by${w}_{i} (i = 1, 2, . . . , m)$. The performance ratings of each decision-maker ${D}_{\mathrm{k}} (k = 1, 2, . . . , K)$ for each alternative $Aj (j = 1, 2, . . . , n)$ with respect to criteria ${C}_{\mathrm{i}} (i = 1, 2, . . . , m)$ are denoted by ${\widetilde{R}}_{\mathrm{k}} = {\widetilde{x}}_{ijk} (i= 1, 2, . . .,m; j= 1, 2, . . ., n; k= 1, 2, . . ., K$ with membership function ${\mu }_{{\widetilde{R}}_{\mathrm{k}}}\left(x\right).$

**Step 2:** Assignment of ratings to the criteria and the alternatives.

Compute aggregate fuzzy ratings for the criteria and the alternatives.

If the fuzzy ratings of all decision-makers are described as triangular fuzzy number${\widetilde{R}}_{\mathrm{k}} =\left({a}_{\mathrm{K}},{b}_{\mathrm{K}},{c}_{\mathrm{K}}\right), k= 1, 2, . . . , K$, then the aggregated fuzzy rating is given by $R =\left(a, b, c\right), k=(1, 2, . . . , K)$ where:

13 $$a=\mathrm{min}\left\{{a}_{k}\right\} , b=\frac{1}{k} \sum_{k=1}^{k}{b}_{k}, c=\mathrm{max}\left\{{c}_{k}\right\}$$

If the fuzzy rating and importance weight of the kth decision-maker are ${\widetilde{x}}_{ijk}= \left({a}_{ijk},{b}_{ij},{c}_{ijk}\right)$ and ${w}_{\mathrm{ijk}}=({ w}_{jk1},{ w}_{jk2},{ w}_{jk3})$; $i =1, 2, . . . ,m; j= 1, 2, . . . n$, respectively, then the aggregated fuzzy ratings $({\widetilde{x}}_{ij})$ of alternatives with respect to each criteria are given by ${x}_{ij} = {a}_{ij},{ b}_{ij}, {c}_{ij}$ where

14 $${a}_{ij}=min\left\{{a}_{ijk}\right\}, {b}_{ij}=\frac{1}{k} \sum_{k=1}^{k}{b}_{ijk}, {c}_{ij}=max\left\{{c}_{ijk}\right\}$$

The aggregated fuzzy weights $({w}_{ij})$ of each criterion are calculated as ${\widetilde{w}}_{j}=({w}_{j1},{w}_{j2},{w}_{j3})$ where:

15 $${w}_{j1}=min\left\{{w}_{jk1}\right\}, {w}_{j2}=\frac{1}{k} \sum_{k=1}^{k}{b}_{jk2}, {w}_{j3}=max\left\{{c}_{jk3}\right\}$$

**Step 3:** Compute the fuzzy decision matrix.

The fuzzy decision matrix for the alternatives $\left(\widetilde{D}\right)$ and the criteria $(\widetilde{W})$ is constructed as follows:

$$\begin{array}{ccc}C1 & C2& C3\end{array}$$

16 $$\widetilde{D}=\begin{array}{c}A1\\ A2\\ A3\end{array}\left[\begin{array}{ccc}{\widetilde{x}}_{11}& \cdots & {\widetilde{x}}_{1n}\\ \vdots & \ddots & \vdots \\ {\widetilde{x}}_{m1}& \cdots & {\widetilde{x}}_{mn}\end{array}\right], i=\left({1,2},..,m\right), j=(\mathrm{1,2},..n)$$

**Step 4:** Normalize the fuzzy decision matrix.

The raw data are normalized using linear scale transformation to bring the various criteria scales into a comparable scale. The normalized fuzzy decision matrix $\widetilde{R}$ is given by:

17 $$\widetilde{R}= {\left[{\widetilde{r}}_{ij}\right]}_{m*n}, , i=\left(\mathrm{1,2},..,m\right), j=(\mathrm{1,2},..n)$$

where:

18 $${\widetilde{r}}_{ij}= \left(\frac{{a}_{ij}}{{C}_{i}^{*}},\frac{{b}_{ij}}{{C}_{i}^{*}},\frac{ {c}_{ij}}{{C}_{i}^{*}}\right) \mathrm{and }{C}_{i}^{*}=\mathrm{max}{c}_{ij} \left(\text{benefit criteria}\right)$$

19 $${\widetilde{r}}_{ij}= \left(\frac{{a}_{j}^{-}}{ {c}_{ij}},\frac{{a}_{j}^{-}}{ {c}_{ij}},\frac{ {a}_{j}^{-}}{ {c}_{ij}}\right) \mathrm{and} {a}_{j}^{-}=\mathrm{max}{c}_{ij} \left(\text{cost criteria}\right)$$

**Step 5:** Compute the weighted normalized matrix.

The weighted normalized matrix $\widetilde{V}$ for criteria is computed by multiplying the weights (${\widetilde{W}}_{j}$) of evaluation criteria with the normalized fuzzy decision matrix ${\widetilde{r}}_{ij}$.

20 $$\widetilde{v}= {\left[{\widetilde{v}}_{ij}\right]}_{m*n}, , i=\left(\mathrm{1,2},..,m\right), j=(\mathrm{1,2},..n)$$

where:

$${\widetilde{v}}_{ij}= {\widetilde{r}}_{ij}*{w}_{j}$$

**Step 6:** Compute the fuzzy positive ideal solution (FPIS) and fuzzy negative ideal solution (FNIS).

The FPIS and FNIS of the alternatives are computed as follows:

21 $${A}^{*}=({\widetilde{v}}_{1}^{*}, {\widetilde{v}}_{2}^{*},\dots , {\widetilde{v}}_{n}^{*})$$

where: $${\widetilde{v}}_{j}^{*}=a{x}_{i}\left\{{v}_{ij3}\right\}, i=\left(\mathrm{1,2},..,m\right), j=(\mathrm{1,2},..n)$$

22 $${A}^{-}=({\widetilde{v}}_{1}^{-}, {\widetilde{v}}_{2}^{-},\dots , {\widetilde{v}}_{n}^{-})$$

where: $${\widetilde{v}}_{j}^{-}={min}_{i}\left\{{v}_{ij1}\right\}, i=\left(\mathrm{1,2},..,m\right), j=(\mathrm{1,2},..n)$$

**Step 7:** Compute the distance of each alternative from FPIS and FNIS.

The distance $({d}_{i}^{*} ,{d}_{i}^{-} )$ of each weighted alternative $i=\left(\mathrm{1,2},..,m\right)$ from the FPIS and the FNIS is computed as follows:

23 $${d}_{i}^{*}=\sum_{j=1}^{n}{d}_{v}\left({\widetilde{v}}_{ij},{\widetilde{v}}_{j}^{*}\right), i=\left(\mathrm{1,2},..,m\right)$$

24 $${d}_{i}^{-}=\sum_{j=1}^{n}{d}_{v}\left({\widetilde{v}}_{ij},{\widetilde{v}}_{j}^{-}\right), i=\left(\mathrm{1,2},..,m\right)$$

where ${d}_{v}\left(\widetilde{a},\widetilde{b}\right)$ is the distance measurement between two fuzzy numbers $\widetilde{a}$ and $\widetilde{b}$.

**Step 8:** Compute the closeness coefficient $(C{C}_{i})$ of each alternative.

The closeness coefficient $(C{C}_{i})$ represents the distances to the fuzzy positive ideal solution $({A}^{*})$ and the fuzzy negative ideal solution (${A}^{-}$) simultaneously. The closeness coefficient of each alternative is calculated as:

25 $$C{C}_{i}= \frac{{d}_{i}^{-}}{{d}_{i}^{-}+{d}_{i}^{*}} , i=\left(\mathrm{1,2},..,m\right)$$

**Step 9:** Rank the alternatives.

In step 9, the different alternatives are ranked according to the closeness coefficient $(C{C}_{i})$ in decreasing order. The best alternative is closest to the FPIS and farthest from the FNIS.

## Results and discussion

In Aleppo, we put our recommended process for choosing a new hospital's location into practice. The problem of deciding where they want to live is still an issue. MCDM faces a huge challenge when it comes to deciding on a location. The fuzzy MCDM approach of "Fuzzy AHP and Fuzzy TOPSIS" was used to solve the problem in this study in a context with uncertainty and ambiguity (fuzziness). The fuzzy TOPSIS method's flow diagram shows the steps as follows:

1. Establishing a committee of decision-makers
2. Determining assessment criteria
3. Determining linguistic variables
4. Applying F-AHP to defining the weight of criteria
5. Forming the fuzzy decision matrix,
6. Forming the normalized fuzzy decision matrix,
7. Forming the weighted normalized fuzzy decision matrix,
8. Determining the values of A* and ${A}^{-}$
9. Calculating the distance of each alternative from A* and ${A}^{-}$
10. Calculating the closeness coefficient of each alternative (${CC}_{i}$),
11. Ranking the alternatives based on their closeness coefficients

**Fig. 3:**
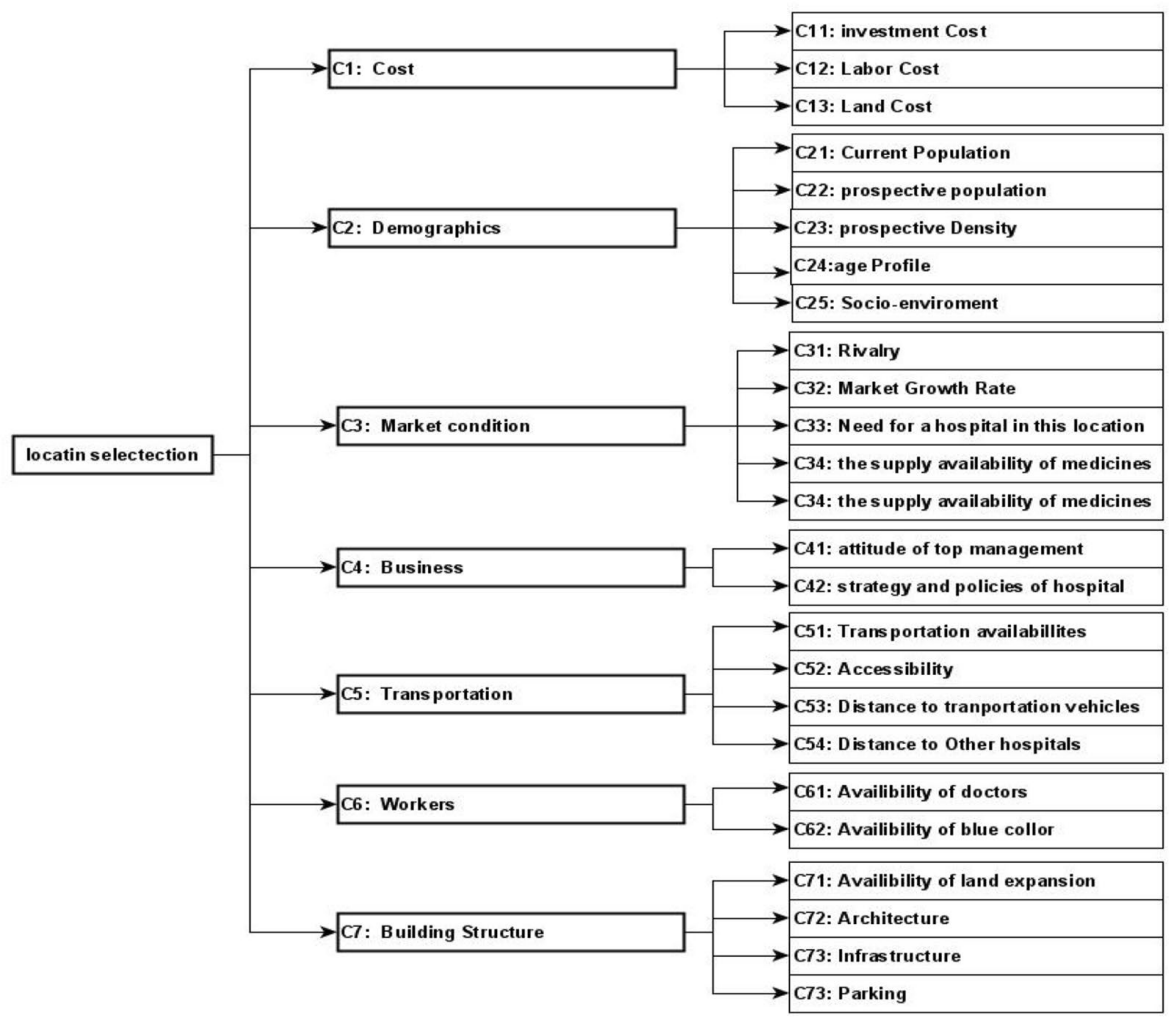
Hierarchy for hospital location selection

These steps are briefly explained below:

**Step 1:** judgments are collected from decision-makers (${D}_{1},{D}_{2},{D}_{3}$) for each criteria and sub-criteria illustrated in Fig. 3.

**Step 2:** For our application research, we primarily investigate four potential sites Al Ezaha (A1), Halab Al Jadidah (A2), Salah Alden (A3), Al Sakhor (A4) regions, were accepted as alternative locations for the location selection problem. At this step, the evaluation criteria are determined. As the determination of the criteria that will be used for evaluation of alternative locations has a direct impact on the accuracy and quality of the results. Relying on previous studies as a study [85] and based on the results of interviews that were conducted with experts, it was determined seven criteria; these seven criteria are Cost (C1), Demographics (C2), Market condition (C3), Business (C4), Transportation (C5) and Workers (C6), Building structure (C7). It can be seen in Table 4 that the C1 criterion is the cost category (C) criteria, that is, the lower the value, the more suitable the alternative. The remaining criteria C2–C7 are the criteria of the type of benefit (B), that is, the higher the value, the better the choice of hospital.

**Table 4:** Criteria for evaluation of  hospital location selection

*Criteria*	*Definition*	*Category*
Cost	expresses the costs incurred by the owner of the facility in terms of workers, investment costs, the cost of buying or renting land, and other costs	**C**
Demographics	It expresses the size of the population, the extent of population density, the age groups in each region, the social environment, and other divisions	**B**
Market condition	It expresses the market expansion, the existing market shares and their percentage, the extent of this market’s need for hospital sites, and the availability of the necessary supplies for the supply of medical medicines and other therapeutic matters	**B**
Business	It expresses the methods followed by the senior management in hospitals, in addition to the strategies and policies adopted by the administration in the hospital management process	**B**
Transportation	It expresses the availability of services and means of transportation in the region, and how close and far the hospital site is from the means of transportation	**B**
Workers	It expresses the availability of human capital and manpower, including doctors and nurses, in the hospital site	**B**
Building structure	It reflects the readiness of the infrastructure and construction, and the availability of service and entertainment places such as parking and a children's park on site	**B**

**Step 3:** This stage determines the proper linguistic variables for assessing both criteria and options, which may be expressed in positive triangular fuzzy numbers. Table 3 shows the linguistic variables used to evaluate the criteria, as well as the triangular fuzzy numbers that corresponded to these linguistic variables, while Table 2 lists the linguistic variables used to evaluate the alternatives, as well as the triangular fuzzy numbers that corresponded to these linguistic variables.

**Step 4:** After choosing the appropriate linguistic variables, decision-makers determine the initial pair-wise comparison matrices as shown in Table 6 by applying F-AHP to defining the weight of criteria and evaluate the ratings of alternative locations with respect to each criterion.

**Table 5:** Linguistic scale of experts

	*C1*	*C2*	*C3*	*C4*	*C5*	*C6*	*C7*		*C1*	*C2*	*C3*	*C4*	*C5*	*C6*	*C7*		*C1*	*C2*	*C3*	*C4*	*C5*	*C6*	*C7*
$C1$	$\widetilde{1}$	$\widetilde{2}$	$\widetilde{1}$	${\widetilde{3}}^{-1}$	$\widetilde{3}$	$\widetilde{2}$	$\widetilde{1}$	$C1$	$\widetilde{1}$	$\widetilde{1}$	$\widetilde{1}$	${\widetilde{2}}^{-1}$	$\widetilde{3}$	$\widetilde{1}$	$\widetilde{2}$	$C1$	$\widetilde{1}$	$\widetilde{1}$	$\widetilde{2}$	${\widetilde{2}}^{-1}$	$\widetilde{2}$	$\widetilde{1}$	$\widetilde{2}$
$C2$	${\widetilde{2}}^{-1}$	$\widetilde{1}$	${\widetilde{2}}^{-1}$	${\widetilde{4}}^{-1}$	$\widetilde{1}$	${\widetilde{3}}^{-1}$	$\widetilde{2}$	$C2$	$\widetilde{1}$	$\widetilde{1}$	${\widetilde{2}}^{-1}$	${\widetilde{2}}^{-1}$	$\widetilde{1}$	${\widetilde{2}}^{-1}$	$\widetilde{1}$	$C2$	$\widetilde{1}$	$\widetilde{1}$	${\widetilde{3}}^{-1}$	${\widetilde{3}}^{-1}$	${\widetilde{2}}^{-1}$	$\widetilde{1}$	${\widetilde{2}}^{-1}$
$C3$	$\widetilde{1}$	$\widetilde{2}$	$\widetilde{1}$	${\widetilde{3}}^{-1}$	$\widetilde{2}$	$\widetilde{2}$	${\widetilde{2}}^{-1}$	$C3$	$\widetilde{1}$	$\widetilde{2}$	$\widetilde{1}$	${\widetilde{4}}^{-1}$	$\widetilde{3}$	$\widetilde{1}$	${\widetilde{2}}^{-1}$	$C3$	${\widetilde{2}}^{-1}$	$\widetilde{3}$	$\widetilde{1}$	${\widetilde{2}}^{-1}$	$\widetilde{3}$	$\widetilde{2}$	$\widetilde{1}$
$C4$	$\widetilde{3}$	$\widetilde{4}$	$\widetilde{3}$	$\widetilde{1}$	$\widetilde{2}$	$\widetilde{4}$	$\widetilde{1}$	$C4$	$\widetilde{2}$	$\widetilde{2}$	$\widetilde{4}$	$\widetilde{1}$	$\widetilde{1}$	$\widetilde{2}$	$\widetilde{3}$	$C4$	$\widetilde{2}$	$\widetilde{3}$	$\widetilde{2}$	$\widetilde{1}$	$\widetilde{3}$	$\widetilde{3}$	$\widetilde{2}$
$C5$	${\widetilde{3}}^{-1}$	$\widetilde{1}$	${\widetilde{2}}^{-1}$	${\widetilde{2}}^{-1}$	$\widetilde{1}$	${\widetilde{2}}^{-1}$	$\widetilde{1}$	$C5$	${\widetilde{3}}^{-1}$	$\widetilde{1}$	${\widetilde{3}}^{-1}$	$\widetilde{1}$	$\widetilde{1}$	$\widetilde{1}$	$\widetilde{2}$	$C5$	${\widetilde{2}}^{-1}$	$\widetilde{2}$	${\widetilde{3}}^{-1}$	${\widetilde{3}}^{-1}$	$\widetilde{1}$	$\widetilde{1}$	${\widetilde{2}}^{-1}$
$C6$	${\widetilde{2}}^{-1}$	$\widetilde{3}$	${\widetilde{2}}^{-1}$	${\widetilde{4}}^{-1}$	$\widetilde{2}$	$\widetilde{1}$	${\widetilde{2}}^{-1}$	$C6$	$\widetilde{1}$	$\widetilde{2}$	$\widetilde{1}$	${\widetilde{2}}^{-1}$	$\widetilde{1}$	$\widetilde{1}$	$\widetilde{1}$	$C6$	$\widetilde{1}$	$\widetilde{1}$	${\widetilde{2}}^{-1}$	${\widetilde{3}}^{-1}$	$\widetilde{1}$	$\widetilde{1}$	$\widetilde{1}$
$C7$	$\widetilde{1}$	$\widetilde{2}$	$\widetilde{2}$	$\widetilde{1}$	$\widetilde{1}$	${\widetilde{2}}^{-1}$	$\widetilde{1}$	$C7$	${\widetilde{2}}^{-1}$	$\widetilde{1}$	$\widetilde{2}$	${\widetilde{3}}^{-1}$	${\widetilde{2}}^{-1}$	$\widetilde{1}$	$\widetilde{1}$	$C7$	${\widetilde{2}}^{-1}$	$\widetilde{2}$	$\widetilde{1}$	${\widetilde{2}}^{-1}$	$\widetilde{2}$	$\widetilde{1}$	$\widetilde{1}$

### Applying F-AHP to defining the weight of criteria

Based on the examination of three decision-makers, the initial pair-wise comparison matrices are created. The linguistic scales are then transformed into a fuzzy number in Table 6 in accordance with Table 2.

**Table 6:** Fuzzy pairwise comparison matrices of criteria

	$C1$			$C2$			$C3$			$C4$		
$C1$	(1.00, 1.00, 1.00)			(1.00, 1.26, 1.44)			(1.00, 1.26, 1.44)			(0.30, 0.44, 0.79)		
$C2$	(0.69, 0.79, 1.00)			(1.00, 1.00, 1.00)			(0.30, 0.44, 0.79)			(0.26, 0.35, 0.55)		
$C3$	(0.69, 0.79, 1.00)			(1.26, 2.29, 3.30)			(1.00, 1.00, 1.00)			(0.26, 0.35, 0.55)		
$C4$	(1.26, 2.29, 3.30)			(1.82, 2.88, 3.91)			(1.82, 2.88, 3.91)			(1.00, 1.00, 1.00)		
$C5$	(0.28, 0.38, 0.63)			(1.00, 1.26, 1.44)			(0.28, 0.38, 0.63)			(0.44, 0.55, 0.79)		
$C6$	(0.69, 0.79, 1.00)			(1.26, 1.82, 2.29)			(0.48, 0.63, 1.00)			(0.26, 0.35, 0.55)		
$C7$	(0.48, 0.63, 1.00)			(0.69, 1.00, 1.44)			(1.00, 1.59, 2.08)			(0.44, 0.55, 0.79)		

	$C5$			$C6$			$C7$		
$C1$	(1.59, 2.62, 3.63)			(1.00, 1.26, 1.44)			(1.00, 1.59, 2.08)		
$C2$	(0.69, 0.79, 1.00)			(0.44, 0.55, 0.79)			(0.69, 1.00, 1.44)		
$C3$	(1.59, 2.62, 3.63)			(1.00, 1.59, 2.08)			(0.48, 0.63, 1.00)		
$C4$	(1.26, 1.82, 2.29)			(1.82, 2.88, 3.91)			(1.26, 1.82, 2.29)		
$C5$	(1.00, 1.00, 1.00)			(0.69, 0.79, 1.00)			(0.69, 1.00, 1.44)		
$C6$	(1.00, 1.26, 1.44)			(1.00, 1.00, 1.00)			(0.69, 0.79, 1.00)		
$C7$	(0.69, 1.00, 1.44)			(1.00, 1.26, 1.44)			(1.00, 1.00, 1.00)		

The synthetic pair-wise comparison matrix of criteria is presented following Formula (3) shown in Table 5.

**Fig. 4:**
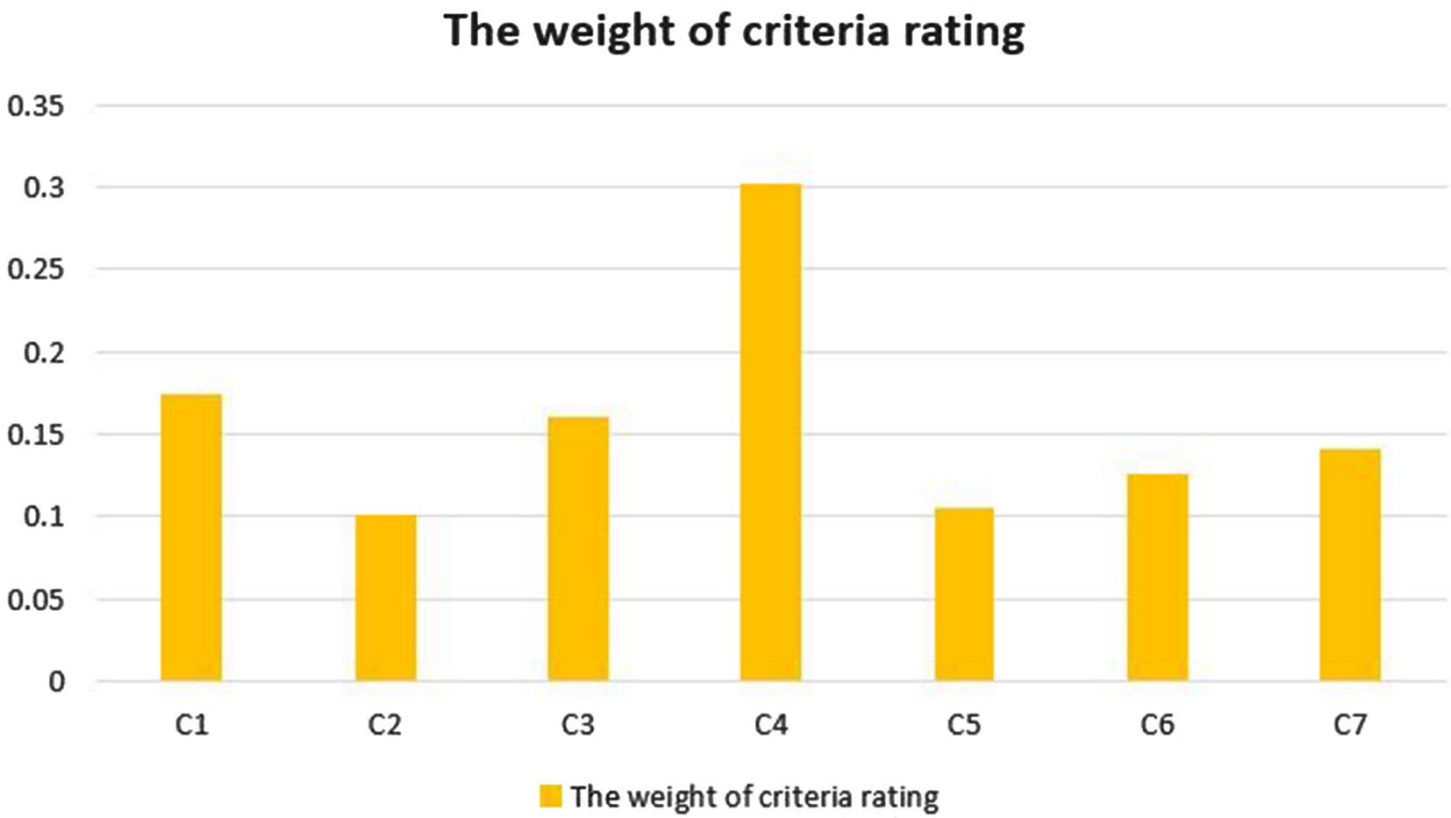
The weight of criteria rating

The fuzzy geometric mean, fuzzy criteria weightage BNP values are demonstrated by applying Formulas (10)–(12), as in Table 7 as shown in the Fig. 4.

**Table 7:** The weight of criteria rating

	Fuzzy geometric mean			Fuzzy weight			BNP	RANK
	A	B	C	A	B	C		
$C1$	0.9007	1.2024	1.5112	0.0914	0.1597	0.2701	0.174	2
$C2$	0.5268	0.6564	0.9057	0.0535	0.0872	0.1619	0.101	7
$C3$	0.7766	1.0742	1.4540	0.0788	0.1427	0.2599	0.160	3
$C4$	1.4262	2.1022	2.6972	0.1447	0.2792	0.4821	0.302	1
$C5$	0.5534	0.6973	0.9414	0.0562	0.0926	0.1683	0.106	6
$C6$	0.6899	0.8479	1.0891	0.0700	0.1126	0.1947	0.126	5
$C7$	0.7207	0.9490	1.2568	0.0731	0.1260	0.2247	0.141	4

**Step 5:** After Applying F-AHP To Defining the Weight of Criteria, the ratings of four alternatives under seven criteria are presented in Table 8.

**Table 8:** Ratings of alternatives by decision-makers under selected criteria

Criteria	Alternatives	Decision-makers		
		D1	D2	D3
C1	A1	G	G	G
	A2	VG	VG	G
	A3	MP	MP	VP
	A4	MP	VP	P
C2	A1	G	G	VG
	A2	F	MP	MP
	A3	G	G	VG
	A4	MG	F	F
C3	A1	F	F	MP
	A2	G	VG	VG
	A3	MP	MP	P
	A4	MP	P	VP
C4	A1	MP	F	MP
	A2	G	G	VG
	A3	MP	MP	P
	A4	P	MP	P
C5	A1	F	F	MG
	A2	G	G	VG
	A3	MP	MP	P
	A4	MP	P	P
C6	A1	G	G	VG
	A2	MG	MG	G
	A3	VG	G	G
	A4	G	G	G
C7	A1	MP	MP	F
	A2	VG	VG	G
	A3	MP	P	MP
	A4	MP	VP	P

**Step 6:** This step involves constructing the fuzzy decision matrix for ranking alternatives as in Table 9 [47, 75].

**Table 9:** Fuzzy decision matrix

Criteria	A1	A2	A3	A4
C1	$(\mathrm{7.00,9.00,10.0})$	$(8.33, 9.66, 10.0)$	$(\mathrm{0.67,2.00,3.67})$	$(\mathrm{0.33,1.33,3.00})$
C2	$(\mathrm{7.67,9.34,10.0})$	$(\mathrm{1.66,3.66,5.66})$	$(\mathrm{7.66,9.33,10.0})$	$(\mathrm{3.66,5.66,7.66})$
C3	$\left(\mathrm{2.33,4.33,6.33}\right)$	$\left(\mathrm{8.33,9.66,10.0}\right)$	$(\mathrm{0.66,2.33,4.33})$	$(\mathrm{0.33,1.33,3.00})$
C4	$(\mathrm{1.66,3.66,5.66})$	$(\mathrm{7.66,9.33,10.0})$	$(\mathrm{0.66,2.33,4.33})$	$(\mathrm{0.33,1.66,3.66})$
C5	$(\mathrm{3.66,5.66,7.66})$	$(\mathrm{7.66,9.33,10.0})$	$(\mathrm{0.66,2.33,4.33})$	$(\mathrm{0.33,1.66,3.66})$
C6	$(\mathrm{7.66,9.33,10.0})$	$(\mathrm{5.66,7.66,9.33})$	$(\mathrm{7.66,9.33,10.0})$	$(\mathrm{7.00,9.00,10.0})$
C7	$(\mathrm{1.66,3.66,5.66})$	$(\mathrm{8.33,9.66,10.0})$	$(\mathrm{0.66,2.33,4.33})$	$(\mathrm{0.33,1.33,3.00})$

**Step 7:** At this step, the raw data are normalized to make comparisons across criteria. The normalized fuzzy decision matrix is constructed as in Table 10.

**Table 10:** Normalized fuzzy decision matrix

Criteria	A1	A2	A3	A4
C1	$(\mathrm{0.700,0.900,1},00)$	$(\mathrm{0.833,0.966}, \mathrm{1,00})$	$(\mathrm{0.067,0.20,0.367})$	$(\mathrm{0.033,0.133,0.30})$
C2	$(\mathrm{0.767,0.934,1},00)$	$(\mathrm{0.166,0.366,0.566})$	$(\mathrm{0.766,0.933,1},00)$	$(\mathrm{0.366,0.566,0.766})$
C3	$\left(\mathrm{0.233,0.433,0.633}\right)$	$\left(\mathrm{0.833,0.966,1},00\right)$	$(\mathrm{0.066,0.233,0.433})$	$(\mathrm{0.033,0.133,0.30})$
C4	$(\mathrm{0.166,0.366,0.566})$	$(\mathrm{0.766,0.933,1},00)$	$(\mathrm{0.066,0.233,0.433})$	$(\mathrm{0.033,0.166,0.366})$
C5	$(\mathrm{0.366,0.566,0.766})$	$(\mathrm{0.766,0.933,1.00})$	$(\mathrm{0.066,0.233,0.433})$	$(\mathrm{0.033,0.166,0.366})$
C6	$(\mathrm{0.766,0.933,1},00)$	$(\mathrm{0.566,0.766,0.933})$	$(\mathrm{0.766,0.933,1.00})$	$(\mathrm{0.700,0.900,1},00)$
C7	$(\mathrm{0.166,0.366,0.566})$	$(\mathrm{0.833,0.966,1},00)$	$(\mathrm{0.066,0.233,0.433})$	$(\mathrm{0.033,0.133,0.300})$

**Step 8:** After the normalized fuzzy decision matrix is constructed, since each criterion has a different importance, the weighted normalized fuzzy decision matrix should be constructed as in Table 11.

**Table 11:** Weighted normalized fuzzy decision matrix

Criteria	A1	A2	A3	A4
C1	$(\mathrm{0.064,0.144,0.269})$	$(\mathrm{0.0769,0.154}, 0.269)$	$(\mathrm{0.006,0.032,0.098})$	$(\mathrm{0.003,0.021,0.081})$
C2	$(\mathrm{0.043,0.086,0.168})$	$(\mathrm{0.009,0.033,0.095})$	$(\mathrm{0.043,0.086,0.168})$	$(\mathrm{0.020,0.052,0.129})$
C3	$\left(\mathrm{0.027,0.042,0.056}\right)$	$\left(\mathrm{0.017,0.032,0.047}\right)$	$(\mathrm{0.061,0.071,0.074})$	$(\mathrm{0.005,0.017,0.032})$
C4	$(\mathrm{0.023,0.095,0.025})$	$(\mathrm{0.105,0.243,0.446})$	$(\mathrm{0.009,0.060,0.193})$	$(\mathrm{0.004,0.043,0.163})$
C5	$(\mathrm{0.188,0.047,0.114})$	$(\mathrm{0.093,0.078,0.149})$	$(\mathrm{0.003,0.019,0.064})$	$(\mathrm{0.001,0.014,0.054})$
C6	$(\mathrm{0.051,0.103,0},192)$	$(\mathrm{0.038,0.085,0.180})$	$(\mathrm{0.051,0.103,0.192})$	$(\mathrm{0.046,0.099,0},192)$
C7	$(\mathrm{0.010,0.039,0.109})$	$(\mathrm{0.051,0.103,0.192})$	$(\mathrm{0.004,0.025,0.083})$	$(\mathrm{0.021,0.014,0.057})$

**Step 9:** At this step, the fuzzy positive ideal solution (A*) and the fuzzy negative ideal solution (${A}^{-}$) are determined as shown below by using the values on the weighted normalized fuzzy decision matrix.

$${A}^{*}=\left[\left(\mathrm{1,1},1\right),\left(\mathrm{1,1},1\right),\left(\mathrm{1,1},1\right),\left(\mathrm{1,1},1\right),\left(\mathrm{1,1},1\right),\left(\mathrm{1,1},1\right),\left(\mathrm{1,1},1\right)\right]$$

$${A}^{-}=\left[\left(\mathrm{0,0},0\right),\left(\mathrm{0,0},0\right),\left(\mathrm{0,0},0\right),\left(\mathrm{0,0},0\right),\left(\mathrm{0,0},0\right),\left(\mathrm{0,0},0\right),\left(\mathrm{0,0},0\right)\right]$$

**Step 10:** The distances of each location alternative from A* and ${A}^{-}$ with respect to each criterion are calculated as in Tables 12 and 13.

**Table 12:** Distances between ${A}_{i}(i=\mathrm{1,2},\mathrm{3,4})$ and A* for each criterion

Criteria	$d (A1, {A}^{*})$	$d (A2,{A}^{*})$	$d (A3,{A}^{*})$	$d (A4, {A}^{*})$
C1	$0.138$	$0.129$	$0.065$	$0.056$
C2	$0.087$	$0.061$	$0.087$	$0.077$
C3	$0.019$	$0.019$	$0.007$	$0.179$
C4	$0.160$	$0.228$	$0.131$	$0.115$
C5	$0.067$	$0.075$	$0.044$	$0.038$
C6	$0.096$	$0.098$	$0.096$	$0.099$
C7	$0.070$	$0.096$	$0.057$	$0.040$

**Table 13:** Distances between ${A}_{i}(i=\mathrm{1,2},\mathrm{3,4})$ and ${A}^{\leftharpoonup }$ each criterion

Criteria	$d (A1, {A}^{\leftharpoonup })$	$d (A2, {A}^{\leftharpoonup })$	$d (A3, {A}^{\leftharpoonup })$	$d (A4, {A}^{\leftharpoonup })$
C1	$0.126$	$0.119$	$0.055$	$0.046$
C2	$0.076$	$0.051$	$0.076$	$0.065$
C3	$0.191$	$0.191$	$0.009$	$0.017$
C4	$0.139$	$0.212$	$0.110$	$0.094$
C5	$0.057$	$0.067$	$0.036$	$0.031$
C6	$0.087$	$0.086$	$0.087$	$0.089$
C7	$0.059$	$0.087$	$0.047$	$0.032$

**Step 11:** Then, the closeness coefficient (${CC}_{i}$) of each location alternative is calculated to determine the ranking order of each alternative as shown in Table 14.

**Table 14:** Calculations of the ${di}^{*}$, ${di}^{\leftharpoonup }$ and closeness coefficient (${CC}_{i}$) values

Criteria	$A1$	$A2$	$A3$	$A4$
${di}^{*}$	$0.639$	$0.709$	$0.488$	$0.446$
${di}^{\leftharpoonup }$	$0.566$	$0.643$	$0.423$	$0.377$
${di}^{*}+{di}^{\leftharpoonup }$	$1.206$	$1.353$	$0.911$	$0.823$
${CC}_{i}$	$0.469$	$0.475$	$0.463$	$0.458$
RANK	2	1	3	4

**Step 12:** Finally, based on the closeness coefficients, the ranking order of the location alternatives can be determined. As shown in Table 14, based on the closeness coefficients (${CC}_{i}$), the ranking order of the location alternatives occurs as follows:

$$\mathrm{A}2>\mathrm{A}1>\mathrm{A}3>\mathrm{A}4$$

To further determine the alternatives' current evaluation statuses based on their proximity coefficients, the closed interval [0, 1] was divided into five equal sub-intervals, and linguistic variables were defined for each sub-interval (Table 15) [16].

**Table 15:** Acceptance conditions

Closeness coefficient	Assessment status
${CC}_{i}$€ [0,0.2]	Unadvisable
${CC}_{i}$ € [0.2,0.4]	Advisable with high risk
${CC}_{i}$ € [0.4,0.6]	Advisable with low risk
$${CC}_{i} $$ € [0.6,0.8]	Acceptable
$${CC}_{i} $$€ [0.8,1.0]	Acceptable and preferable

**Fig. 5:**
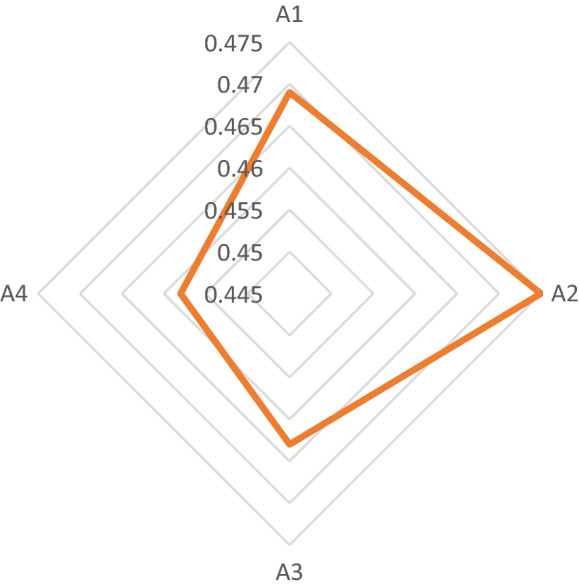
Closeness coefficient (${CC}_{i}$) values

According to Tables 14 and 15, all the alternative sites were found advisable with low risk. Alternative A2 with the highest value (0.512) is the best appropriate alternative location among others. It is followed by A1 and A3 with the values of 0.506 and 0.500, respectively. However, alternative A4 is the worst location for hospital location selection problem (Fig. 5).

## Sensitivity analysis:

A graphical representation of sensitivity analysis depicts the fluctuations in the result as a result of changing input data. The supplier's weight on the criteria in this study is raised from extremely low to good. Depending on the weight of the criterion, different possibilities are ranked in a different order. There are 12 cases that are taken into account. To demonstrate the sensitivity analysis performed using fuzzy TOPSIS, a radar diagram has been created.

**Fig. 6:**
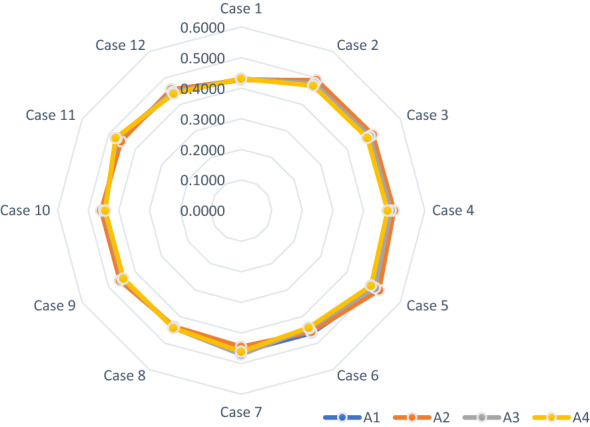
Results of sensitivity analysis

Method as shown in Fig. 6. The radar diagram shows the closeness coefficient of each Site at 12 different cases. If there is any change in criteria weight of supplier, then its outcome will alter in terms of proximity coefficient of suppliers. In all 10 separate situations the proximity coefficient for all suppliers has been computed and is displayed in Fig. 6. Additionally, it is evident in Table 16 and Fig. 6. The option A4 (Site 4) has the highest score in 8 out of the 12 cases, whereas A1 (Site 1) has the lowest score in 8 out of the 12 cases, and A2 (Site 2) has the lowest score in 7 out of the 12 cases.

**Table 16:** Changes to the criteria weights for sensitivity analysis

Case no	Criteria weight changes	Overall scores $(\mathrm{CCr})$				Ranking
		$\mathrm{A}1$	$\mathrm{A}2$	$\mathrm{A}3$	$\mathrm{A}4$	
Case 1	${w}_{c1-c7}=(\mathrm{0.1,0.1,0.3})$	0.4294	0.4269	0.4306	0.4312	$A4>A3>A1>A2$
Case 2	${w}_{c1-c7}=(\mathrm{0.1,0.3,0.5})$	0.4863	0.4924	0.4798	0.4712	$A2>A1>A3>A4$
Case 3	${w}_{c1-c7}=(\mathrm{0.3,0.5,0.7})$	0.4909	0.4966	0.4852	0.4758	$A2>A1>A3>A4$
Case 4	${w}_{c1-c7}=(\mathrm{0.5,0.7,0.9})$	0.4942	0.4997	0.4889	0.4787	$A2>A1>A3>A4$
Case 5	${w}_{c1-c7}=(\mathrm{0.7,0.9,1.0})$	0.5116	0.5204	0.5047	0.4904	$A2>A1>A3>A4$
Case 6	${w}_{c1}=\left(\mathrm{0.7,0.9,1.0}\right),$ ${w}_{c2-c7}=(\mathrm{0.1,0.1,0.3})$	0.4617	0.4573	0.4447	0.4415	$A1>A2>A3>A4$
Case 7	${w}_{c2}=\left(\mathrm{0.7,0.9,1.0}\right),$ ${w}_{c1, c3-c7}=(\mathrm{0.1,0.1,0.3})$	0.4615	0.4448	0.4703	0.4609	$A3>A1>A4>A2$
Case 8	${w}_{c3}=\left(\mathrm{0.7,0.9,1.0}\right),$ ${w}_{c1-2, c4-c7}=(\mathrm{0.1,0.1,0.3})$	0.4405	0.4378	0.4426	0.4431	$A4>A3>A1>A2$
Case 9	${w}_{c4}=\left(\mathrm{0.7,0.9,1.0}\right),$ ${w}_{c1-c3,c5- c7}=(\mathrm{0.1,0.1,0.3})$	0.4479	0.4576	0.4472	0.4444	$A2>A1>A3>A4$
Case 10	${w}_{c5}=\left(\mathrm{0.7,0.9,1.0}\right),$ ${w}_{c1-4, c6-7}=(\mathrm{0.1,0.1,0.3})$	0.4521	0.4576	0.4472	0.4444	$A2>A1>A3>A4$
Case 11	${w}_{c6}=\left(\mathrm{0.7,0.9,1.0}\right),$ ${w}_{c1-5, c7}=(\mathrm{0.1,0.1,0.3})$	0.4615	0.4535	0.4703	0.4740	$A4>A3>A2>A1$
Case 12	${w}_{c7}=\left(\mathrm{0.7,0.9,1.0}\right),$ ${w}_{c1-6}=(\mathrm{0.1,0.1,0.3})$	0.4479	0.4573	0.4472	0.4415	$A2>A1>A3>A4$

## Conclusions

In this paper, we present a multi-criteria decision-making approach to evaluate the four sites for choosing the best alternative in a situation of ambiguity based on the combination of two fuzzy AHP and fuzzy TOPSIS methods. Three steps make up the suggested strategy. The first step identified the four site evaluation criteria. Cost, demography, market situation, business, transportation and labor, and building structure are the seven criteria. The second step involves the specialists giving language evaluations of the standards and options. The ordered weighting of criteria was obtained based on the fuzzy AHP calculation, and to get an overall performance score and compile ratings, the priority ranking of the alternative locations was found using the fuzzy TOPSIS used to assess each alternative's viability. The solution with the greatest score is chosen as the ideal location and is suggested for use in the city. We conduct a sensitivity analysis in the third and final step to ascertain the impact of the weights of the criteria on the decision-making procedure.

The strength of our approach lies in its capacity to evaluate the viability of potential sites while working with sparse or inaccurate data. The regions in the city of Aleppo can use the suggested method practically to assess and choose the best site out of the suggested sites. They must be carefully chosen because the decision-making process is sensitive to the number of participants and their level of subject matter expertise.

We conduct a sensitivity analysis in the third and final step to ascertain the impact of the weights of the criteria on the decision-making procedure. Our method's strength lies in its capacity to evaluate the viability of potential sites while working with sparse or inaccurate data. The regions in the city of Aleppo can use the suggested method practically to assess and choose the best site out of the suggested sites. They must be carefully chosen because the decision-making process is sensitive to the number of participants and their level of subject matter expertise.

Method's strength lies in its capacity to evaluate the viability of potential sites while working with sparse or inaccurate data. The regions in the city of Aleppo can use the suggested method practically to assess and choose the best site out of the suggested sites. They must be carefully chosen because the decision-making process is sensitive to the number of participants and their level of subject matter expertise; it can be also applied to the selection of an optimal location for related healthy projects, such as the location of nurses and doctors, hi-tech medical areas, and factories that producing medical drugs.

Future studies should consider new assessment factors with respect to the post-COVID-19 pandemic that can impact the process of evaluation and selection of locations to enhance the robust results. Besides, other multi-criteria decision-making approaches, such as GIS Based Location Selection, hesitant fuzzy VIKOR, might be used to investigate alternate locations and compare the results to those found in this study. In addition, the accuracy and reliability of these rankings should be measured using a reference for ranking similarity coefficients (i.e., weighted Spearman’s rank correlation coefficient, rank similarity coefficient).

## Data Availability

This is available on request.
